# Clinically important improvement thresholds for Harris Hip Score and its ability to predict revision risk after primary total hip arthroplasty

**DOI:** 10.1186/s12891-016-1106-8

**Published:** 2016-06-10

**Authors:** Jasvinder A. Singh, Cathy Schleck, Scott Harmsen, David Lewallen

**Affiliations:** Department of Medicine, Medicine Service, Birmingham VA Medical Center, University of Alabama, Faculty Office Tower 805B, 510 20th Street S, Birmingham, AL 35294 USA; Departments of Orthopedic Surgery, Mayo Clinic School of Medicine, Rochester, MN USA; Departments of Biostatistics, Mayo Clinic School of Medicine, Rochester, MN USA; Center for Surgical Medical Acute care Research and Transitions (C-SMART), Birmingham VA Medical Center, Birmingham, AL USA; Division of Epidemiology, School of Public Health, University of Alabama, Birmingham, AL USA

**Keywords:** Harris Hip Score, Total Hip Arthroplasty, Responsiveness, Discriminant ability, Predictability, Clinically important improvement, Minimal clinically important improvement, MCII, Minimal clinically important difference, MCID

## Abstract

**Background:**

Some aspects of validity are missing for the Harris Hip Score (HHS). Our objective was to examine the clinically meaningful change thresholds, responsiveness and the predictive ability of the HHS questionnaire.

**Methods:**

We included a cohort of patients who underwent primary total hip arthroplasty (THA) and responded to the HHS preoperatively and at 2- or 5-year post-THA (change score) to examine the clinically meaningful change thresholds (Minimal clinically important improvement, MCII; and moderate improvement), responsiveness (effect size (ES) and standardized response mean (SRM)) based on pre- to post-operative change and the predictive ability of change score or absolute postoperative score at 2- and 5-years post-THA for future revision.

**Results:**

Two thousand six hundred sixty-seven patients with a mean age of 64 years completed baseline HHS; 1036 completed both baseline and 2-year HHS and 669 both baseline and 5-year HHS. MCII and moderate improvement thresholds ranged 15.9–18 points and 39.6–40.1 points, respectively. ES was 3.12 and 3.02 at 2- and 5-years; respective SRM was 2.73 and 2.52. There were 3195 hips with HHS scores at 2-years and 2699 hips with HHS scores at 5-years (regardless of the completion of baseline HHS; absolute postoperative scores). Compared to patients with absolute HHS scores of 81–100 (score range, 0–100), patients with scores <55 at 2- and 5-years had higher hazards (95 % confidence interval) of subsequent revision, 4.34 (2.14, 7.95; *p* < 0.001) and 3.08 (1.45, 5.84; *p* = 0.002), respectively. Compared to HHS score improvement of  >50 points from preoperative to 2-years post-THA, lack of improvement/worsening or 1–20 point improvement were associated with increased hazards of revision, 18.10 (1.41, 234.83; *p* = 0.02); and 6.21 (0.81, 60.73; *p* = 0.10), respectively.

**Conclusions:**

HHS is a valid measure of THA outcomes and is responsive to change. Both absolute HHS postoperative scores and HHS score change postoperatively are predictive of revision risk post-primary THA. We defined MCID and moderate improvement thresholds for HHS in this study.

## Background

Total Hip Arthroplasty (THA) is the second most commonly performed arthroplasty in the U.S. In 2010, 438,000 THAs were done in the U.S. [1] and its utilization rate is increasing rapidly. U.S. Medicare spent $9 billion on implantable medical device procedures in 2009 [2].

The improvements in pain and function after THA are measured with instruments such as Harris Hip Score (HHS) [3]. HHS is the most commonly used instrument for the assessment of outcomes post-THA [4]. HHS is valid and reliable [5–8] and is often used as a reference/gold standard for assessing the construct validity of other patient-reported outcome measures (PROs) for hip outcomes [9]. HHS is more responsive than the Western Ontario McMaster Osteoarthritis Index (WOMAC) [8] (a pain and function composite measure), short form-36 (SF-36) [8, 10, 11] (a generic health-related quality of life measure) and the walking speed [11] (an objective measure). HHS is joint-specific, measures hip outcomes and is widely available. A surgeon or a health professional usually completes HHS.

To our knowledge, despite its widespread use, there are no published data regarding what is a clinically important difference on the HHS or whether the HHS scores are predictive of the risk of future revision surgery. Defining clinically important thresholds for outcomes instruments such as HHS is critical, since it has direct clinical care and clinical trial relevance. For clinical care, this threshold could define what proportion of patients improved (by what matters to a patient, which is more relevant than a mean change for a population and/or a p-value for group means) with any new clinical initiative. For a clinical trial, thresholds would allow a comparative assessment of one intervention vs. another, and thus allow the design of clinical trials with an adequate sample size to differentiate two interventions [12]. If HHS can predict the risk of early revision surgery, future studies could assess its utility as a screening tool for implant failures after THA. The objective of this study was to define the clinically meaningful thresholds for improvement and assess the responsiveness of HHS and examine its predictive ability for early revision surgery in patients with primary THA.

## Methods

### Study participants and the HHS questionnaire

The Human Ethics Committee at the Mayo Clinic approved the study and research was carried out in compliance with the Helsinki Declaration. We received a waiver of written informed patient consent for this database study. Two study cohorts of patients who underwent primary THA at the Mayo Clinic between 1993 and 2005 were examined: (1) patients who completed HHS at baseline and at follow-up at either 2- or 5-year (cohort 1); (2) patients who completed follow-up HHS at either 2- or 5-year (cohort 2). All analyses were performed for the first cohort and in addition, we performed analysis of predictive ability of final postoperative HHS scores at 2- and 5-years for the latter cohort. HHS is a composite measure, with score ranging from 0 to 100, heavily weighted by pain and function; a higher score is better. It includes four domains: pain (1 item; 44 points), physical function (7 items; 47 points), deformity (5 items; 5 points), and range of motion (5 items; 4 points) [3].

### Responsiveness and predictive ability

Minimal clinically important improvements (MCII) and moderate improvements were calculated by assessing mean change from baseline to 2- and 5-year follow-up in patients who reported “somewhat better now” or “much better now” in response to the global question, “Compared to before surgery, how is your hip?” at both 2- and 5-years, respectively. To assess responsiveness, we calculated effect size (ES) and standardized response mean (SRM). We calculated ES by dividing the change in hip score from baseline to 2-years (or 5-years) by the baseline standard deviation (preoperative; SD). According to the Cohen’s rule, an ES of 0.20–0.49 represents a small change, 0.50–0.79 a medium change, and ≥ 0.80 a large change. The SRM is the mean change in the patient score divided by the SD of the changed scores [13]. These analyses were performed for the cohort with pre-operative and at least one post-operative follow-up, 2 or 5-year.

Descriptive statistics were reported as number (percentage) or mean (SD) as appropriate. We examined the associations of the final HHS score at 2- or 5-years or change in HHS score from baseline to 2- and 5-years, with the risk of subsequent revision THA, at ≥ 731 days post-surgery for 2-year and ≥ 1826 days for 5-year (Day 0 for all revision analyses, respectively). Final HHS scores were categorized into ≤ 55, 56–63, 64–71, 72–80 and 81–100, based on quintiles. Only the first hip in a patient was included for this study; patients with simultaneous bilateral THAs were excluded. We performed sensitivity analyses by using the traditional categorization of HHS: < 70, poor; 70–79, fair; 80–89, good; and 90–100 excellent. Improvement in HHS was categorized a *priori* as ≤ 0 (no improvement or worsening), 1–20, 21–50 vs. > 50, based on clinical judgment from an orthopedic surgeon (D.G.L), a co-author of the current study. Cox proportional hazards regression was used, reporting a hazard ratio and 95 % confidence intervals. Kaplan-Meier survival was used to estimate implant survival based on the absolute and change in HHS scores at 2- or 5-years. A *p*-value of less than 0.05 was considered significant. Since death is a competing risk, we also performed competing risk models adjusting for death.

## Results

### Cohort characteristics

**Fig. 1:**
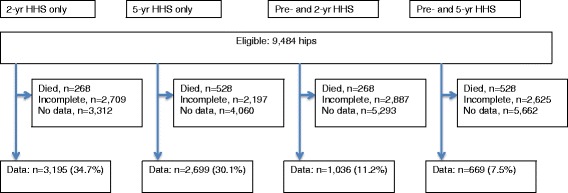
Patient selection for cohorts requiring only 2/5-year HHS scores and those requiring both preoperative and 2/5-year scores

Two thousand six hundred sixty-seven patients had completed baseline HHS, of whom 1036 had completed both baseline and 2-year HHS and 669 had completed both baseline and 5-year HHS; 338 patients completed all three assessments (baseline and 2- and 5-year HHS (Fig. 1). Mean age was 64 years and 51 % were women. The dempgraphic and clinic characteristics of two cohorts used for (1) responsiveness, clinically important improvement thresholds and change HHS scores (both pre- and 2- or 5-year post-arthroplasty scores) and (2) predictive ability of 2- or 5-year HHS scores only, are shown in Appendix: Table 3. The causes for early revision in the 2- and 5-year cohorts are shown in Appendix: Table 4.

### Clinically important improvement thresholds for the HHS and its responsiveness

MCII threshold for HHS ranged 15.9–18 points (Table 1); and moderate improvement threshold was 39.6–40.1 points. We found that the effect sizes for HHS pre- to post-THA were large, estimated at 3.12 and 3.02 at 2-, and 5-years. SRM was 2.73 and 2.52, at 2- and 5-years, respectively (Table 1).

**Table 1:** Responsiveness of the Harris Hip Score (HHS) and thresholds for clinically important differences on the HHS

	2-year - Baseline value [#patients]	5-year - Baseline value [#patients]
Responsiveness (Discriminant Ability)		
Effect Size (ES)	3.12 [*n* = 1036]	3.02 [*n* = 669]
Standardized Response Mean (SRM)	2.73 [*n* = 970]	2.52 [*n* = 637]
Clinically Important Improvement, Mean (Standard deviation)		
Minimal Clinically important Improvement (MCII)	18.0 (18.2) [*n* = 59]	15.9 (19.8) [*n* = 39]
Moderate improvement	40.1 (12.8) [*n* = 911]	39.6 (13.5) [*n* = 598]

### Predictive ability of the absolute and change HHS scores

There were 3195 hips with HHS scores at 2-years and 2699 hips with HHS scores at 5-years (regardless of the completion of the baseline HHS). Low total HHS scores at 2- and 5-years were associated with a higher risk of revision surgery after each time-point (Table 2). Compared to patients with follow-up HHS scores of 81–100, patients with HHS scores < 55 at 2- and 5-years, had 4.34 and 3.08 higher hazards of revision subsequently, both statistically significant (Table 2). Results were similar when we used the traditional clinical cut offs of < 70, 70–79, 80–89, and 90–100 (Table 2).

**Table 2:** Predictive ability of HHS: Association of HHS scores at 2- and 5-years with the risk of revision in patients with THA

	Hazards of THA Revision (95 % CI)	*P*-value*	Hazards of THA Revision (95 % CI)	*P*-value*
	2-year data		5-year data	
	*N* = 3151 with 88 events		*N* = 2461 with 71 events	
Harris Hip Score categories				
≤ 55 vs. 81–100	4.34 (2.14, 7.95)	< 0.001	3.08 (1.45, 5.84)	0.002
56–63 vs. 81–100	1.31 (0.36, 3.32)	0.62	1.25 (0.34, 3.20)	0.69
64–71 vs. 81–100	1.80 (0.59, 4.18)	0.23	0.64 (0.07, 2.36)	0.60
72–80 vs. 81–100	0 (0, 0.5)	0.006	1.13 (0.37, 2.66)	0.80
Harris Hip Score clinical categories				
< 70 vs. 90–100	2.32 (1.32, 3.85)	0.002	1.60 (0.84, 2.85)	0.14
70–79 vs. 90–100	0 (0, 0.5)	0.005	1.18 (I0.38, 2.79)	0.74
80–89 vs. 90–100	0.67 (0.33, 1.23)	0.23	0.66 (0.30, 1.30)	0.27
	*N* = 1011 with 13 events**		*N* = 583 with 5 events**	
Improvements in Harris Hip Score				
≤ 0 vs. 51–75	18.10 (1.41, 234.8)	0.02	2.20 (0.02, 29.01)	0.68
1–20 vs. 51–75	6.21 (0.81, 60.73)	0.10	0.71 (0.005, 8.78)	0.85
21–50 vs. 51–75	2.19 (0.50, 20.55)	0.41	0.62 (0.12, 3.92)	0.64

*P-value was calculated using a Cox proportional hazards regression model

**The main reason for decrease in the number of patients between absolute score (top) and change HHS score analyses (bottom) was that a lower number completed both surveys (See Fig. 1 for details)

**Fig. 2:**
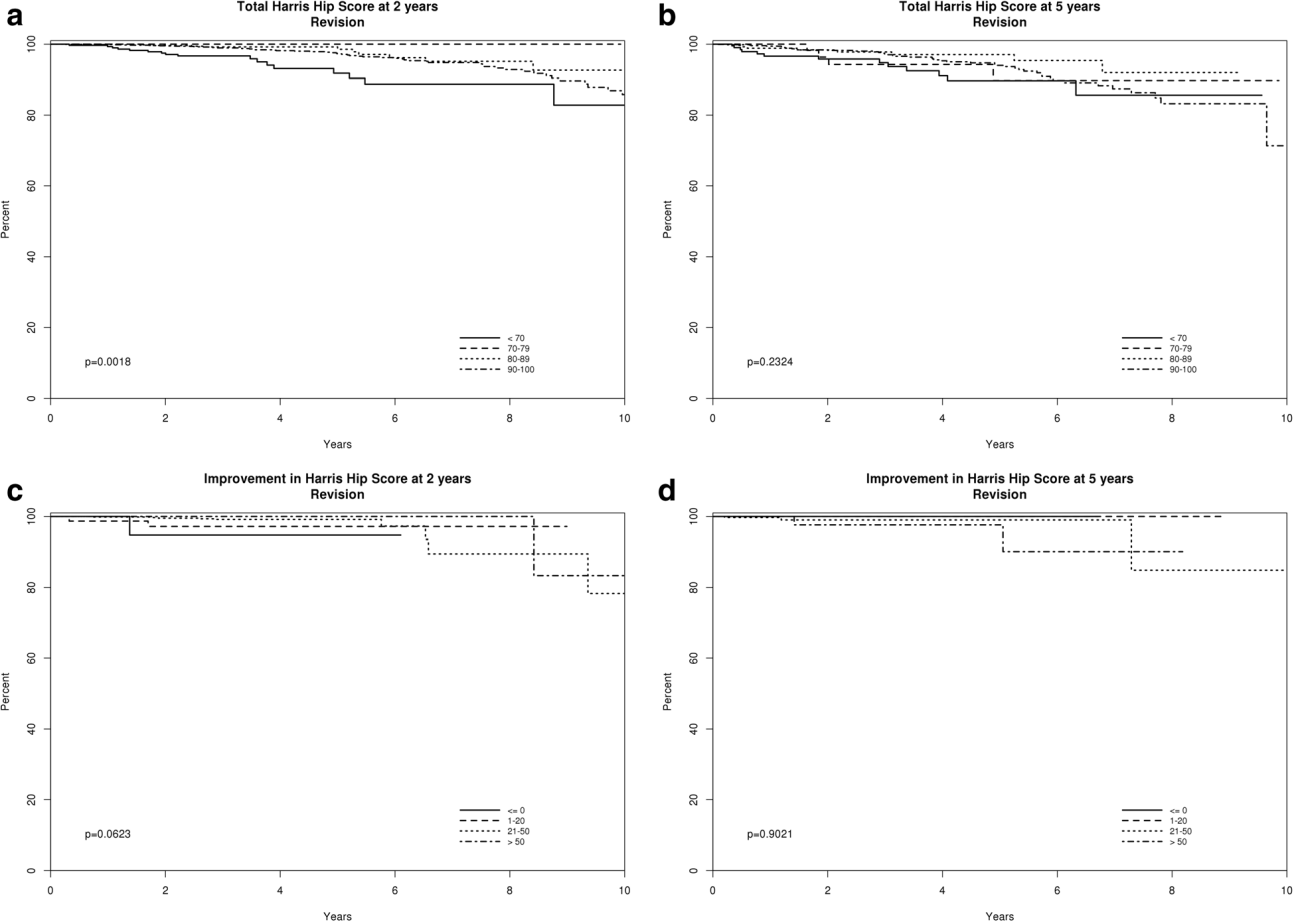
Implant survival is shown for different categories of absolute (panels **a** and **b**) or change HHS scores (panels **c** and **d**), with follow-up up to 10 years post-THA. Follow-up starts at ≥ 731 days post-surgery for 2-year and ≥ 1826 days for 5-year (Day 0 for all revision analyses), i.e., only after the administration of the 2- and 5-year HHS survey

Compared to an improvement of > 50 points, no improvement/worsening HHS score, i.e., change ≤ 0, was associated with a 18-fold increased risk (*p* = 0.02) at 2-years post-primary THA and improvement of only 1–20 points with a 6-fold increased risk of revision (*p* = 0.10) (Table 2). K-M graphs showed that 2-year HHS scores (p =0.0018) was associated with revision risk and change HHS score at 2-years seemed to be associated as well with borderline statistical significance (*p* =0.062; Fig. 2a-d). Models accounting for death as a competing risk confirmed findings; the total number of patients was small for improvement (Appendix: Table 5).

## Discussion

Our study is the first study to define the thresholds for patient-relevant meaningful improvements on HHS. These thresholds were 16–18 points for MCII and 40 points for moderate improvement. We used a patient anchor to define these thresholds. MCII for HHS can now be used for trial sample size calculations and comparative effectiveness studies in THA. The use of MCII in arthroplasty trials will allow future trials to be adequately powered to examine group-level differences in patient-level outcome assessment on HHS. This information will compliment the mean HHS score comparisons, an approach that combines responders and non-responders in a single group. Consensus recommendations from an international pain trial group are to report both means and %responders in clinical trials, where pain is one of the primary outcomes [14]. Similar thresholds have been reported for another instrument, the Mayo Hip Score [15], that is patient-administered, not physician-reported.

Our MCII threshold is similar to/lower than MCII estimates for other 0–100 pain scales, for example, the thresholds of 15–20 mm on WOMAC pain [16], 24 mm on HOOS pain [17], 20 mm on VAS pain in pain trials [18] and 22 mm on VAS pain in gout trials [19]. This finding establishes the sensitivity to change for HHS, an important property for an instrument. Moderate improvement reported in our study are similar to the substantial improvement of 50 % in pain [14, 18] and to American College of Rheumatology (ACR50) response in patients with rheumatoid arthritis, i.e., 50 % improvement in composite criteria [20]. An earlier study in revision THA that did not use a patient anchor defined the MCII using a statistical definition and reported it to be 2.44 points [10], much lower than the 20-unit threshold previously defined on 0–100 pain scales in trials of chronic pain [18] and gout [19]. This might be due to very low variability on HHS in this small sample, and is discordant with available literature for other similar pain scales. A 2 mm improvement on a 0–100 scale (~2–4 % change depending on the baseline) does not mean much to patients or providers, and in most instances will be indistinguishable from baseline noise.

We found that HHS scores < 55 at 2- and 5-years as well as the lack of improvement or worsening on change HHS scores from baseline to 2-years were each predictive of early revision in patients with primary THA. This is a new finding, to our knowledge. The robustness of this finding is supported by the consistency of estimates at both 2- and 5-years. Our finding links HHS at early follow-up to future implant failure and indicates that absolute HHS scores and its trajectory post-THA may help to screen early implant failures after primary THA. More research is needed to see if risk prediction models using PROs such as HHS can be developed for early implant failure and validated, similar to a proposal based on Oxford scores [21].

Wright et al. reported ES and SRM of 2.5 and 1.8 for HHS in a sample of 78 THA patients at 6-month post-surgery [8], confirmed in our study and extended to a longer follow-up. A previous study showed a much larger effect size of 8.6 for HHS in patients with revision THA [10]; SRM in hip fracture was 0.75 for HHS [22]. Some reasons for the difference in findings from our study vs. previous study for HHS effect size were the difference in patient population (primary THA vs. revision THA), length of follow-up (up to 5-years vs. 6 months), and the setting (USA vs. China).

Our study strengths include a large primary THA patient sample from a Joint Registry, the use of well-accepted methods to examine validity, the performance of sensitivity analyses and robustness of estimates across 2- and 5-year data.

Our study has several limitations. MCII thresholds are not absolute, but rather estimates and can vary somewhat from one population to another, and between studies. Our findings were derived from a single center primary THA cohort, and our patient characteristics are similar to several other THA studies including the one with a national sample [23–25], implying that our sample is representative of primary THA. However, findings may not be generalizable to revision THA. However, more studies in other populations can determine the slight variation in MCII thresholds or confirm that these thresholds hold true for those groups as well. Incorporation of 2- or 5-year HHS scores into surgeon’s clinical decision-making may have biased findings to be more significant that they actually might be. However, many revisions occurred at a time much after the assessments, and it is possible that the surgeon did not access old scores. We recognize that radiographic changes after arthroplasty are very important in deciding whether revision surgery is needed or not, since asymptomatic patients sometimes show loose hip implants. The present study did not assess the predictive ability of hip radiographs alongside Harris Hip Score. This should be investigated in future studies. Our study establishes the thresholds for clinically meaningful change on HHS; however, ceiling effects have been noted with HHS [26].

## Conclusions

In conclusion, we found that in patients who underwent primary THA, HHS was responsive to change and predictive of the risk of revision after primary THA. Clinically important improvement thresholds for minimal and moderate clinically important improvements on HHS were defined in this study can now be used in arthroplasty clinical trials and clinical care. This report also establishes an additional utility of HHS, in predicting early revision surgery after THA, an important outcome.

## Abbreviations

CI, confidence interval; HHS, Harris Hip Score; MCID, Minimal clinically important difference; MCII, Minimal clinically important improvement; SD, standard deviation; THA, Total hip arthroplasty

## Appendix

**Table 3:** Demographics of the study populations

	Hips with HHS preoperatively (*n* = 2667)	Hips with HHS preoperative and 2 years (*n* = 1036)	Hips with HHS preoperative and 5 years (*n* = 669)
Age in years, Mean (SD)	64 (14)	65 (13)	64 (13)
Male, *n* (%)	1315 (49.3 %)	501 (48.4 %)	317 (47.4 %)
Age Groups, n (%)			
< = 60 years	889 (33.3 %)	316 (30.5 %)	224 (33.5 %)
61–70 years	754 (28.3 %)	291 (28.1 %)	196 (29.3 %)
71–80 years	894 (30.2 %)	325 (31.4 %)	206 (30.8 %)
≥ 81 years	220 (8.2 %)	104 (10.0 %)	43 (6.4 %)
Body Mass Index^a^, *n* (%)			
< 25 kg/m^2^	587 (22.2 %)	215 (20.8 %)	160 (24.1 %)
25–29.9 kg/m^2^	1032 (38.9 %)	414 (40.2 %)	258 (38.8 %)
30–34.9 kg/m^2^	646 (24.4 %)	262 (25.4 %)	159 (23.9 %)
35–39.9 kg/m^2^	235 (8.9 %)	92 (8.9 %)	56 (8.4 %)
≥ 40 kg/m^2^	150 (5.7 %)	48 (4.7 %)	32 (4.8 %)
American Society of Anesthesiologists Class (ASA)^b^, *n* (%)			
Class I-II	1654 (62.1 %)	645 (62.4 %)	444 (66.5 %)
Class II-IV	1008 (37.9 %)	388 (37.6 %)	224 (33.5 %)
Deyo-Charlson Index, mean (SD)	1.1 (2.0)	1.1 (1.9)	1.0 (1.9)
Psychological Comorbidity, *n* (%)			
Anxiety	135 (5.1 %)	47 (4.5 %)	30 (4.5 %)
Depression	231 (8.7 %)	78 (7.5 %)	50 (7.5 %)
Total number of Joints Replaced			
1	2159 (81.0 %)	824 (68.5 %)	525 (78.5 %)
2	463 (17.4 %)	194 (18.7 %)	135 (20.2 %)
3	38 (1.4 %)	15 (1.4 %)	6 (0.9 %)
4	7 (0.3 %)	3 (0.3 %)	3 (0.4 %)
Underlying Diagnosis, *n* (%)			
Osteoarthritis	2404 (90.1 %)	952 (91.9 %)	598 (89.4 %)
RA/Inflammatory Arthritis	49 (1.8 %)	18 (1.7 %)	14 (2.1 %)
Other	214 (8.0 %0	66 (6.4 %)	57 (8.5 %)

Missing data for preoperative, preoperative and 2-year and preoperative and 5-year HHS cohorts were as follows

^a^For BMI, there were 17, 5 and 4 missing cases, respectively

^b^For ASA, there were 5, 3 and 1 missing cases, respectively

**Table 4:** Causes for early revision in patients with HHS data

	2-years cohort	5-years cohort
	*N* = 403 revisions	*N* = 282 revisions
Aseptic loosening	197	145
Infection	13	5
Dislocation/fracture	107	71
Wear/osteolysis	174	135
Pain	4	2
Other	27	17

**Table 5:** Competing risk-adjusted Models for revision risk adjusting for risk of death

	Hazards of THA Revision (95 % CI)	*P*-value*	Hazards of THA Revision (95 % CI)	*P*-value*
	2-year data		5-year data	
	*N* = 3151 with 88 events		*N* = 2461 with 71 events	
Harris Hip Score categories				
≤ 55 vs. 81–100	3.90 (2.67,5.69)	< 0.001	2.48 (1.56,3.95)	< 0.001
56–63 vs. 81–100	2.24 (1.45,3.46)	< 0.001	2.04 (1.25,3.32)	< 0.001
64–71 vs. 81–100	2.36 (1.60,3.49)	< 0.001	2.78 (1.72,4.48)	< 0.001
72–80 vs. 81–100	2.18 (1.61,2.94)	< 0.001	2.43 (1.70,3.460	< 0.001
Harris Hip Score clinical categories				
< 70 vs. 90–100	3.40 (2.59,4.45)	< 0.001	2.65 (1.92,3.66)	< 0.001
70–79 vs. 90–100	2.66 (1.92,3.68)	< 0.001	2.88 (1.96,4.25)	< 0.001
80–89 vs. 90–100	2.02 (159,2.57)	< 0.001	1.64 (1.21,2.24)	< 0.001
	*N* = 1011 with 13 events		*N* = 583 with 5 events	
Improvements in Harris Hip Score				
≤ 0 vs. 51–75	2.48 (1.00,6.14)	0.05	2.30 (0.46,11.61)	0.31
1–20 vs. 51–75	0.99 (0.38,2.57)	0.98	1.97 (0.63,6.15)	0.24
21–50 vs. 51–75	0.68 (0.40,1.15)	0.15	0.92 (0.42,2.02)	0.84
